# A systematic review of medicinal plants used against *Echinococcus granulosus*

**DOI:** 10.1371/journal.pone.0240456

**Published:** 2020-10-13

**Authors:** Rehman Ali, Sanaullah Khan, Marina Khan, Muhammad Adnan, Ijaz Ali, Taj Ali Khan, Sumbal Haleem, Muhammad Rooman, Sadia Norin, Shahid Niaz Khan

**Affiliations:** 1 Department of Zoology, Kohat University of Science and Technology, Kohat, Khyber Pakhtunkhwa, Pakistan; 2 Department of Zoology, University of Peshawar, Peshawar, Khyber Pakhtunkhwa, Pakistan; 3 Department of Botanical and Environmental Sciences, Kohat University of Science and Technology, Kohat, Khyber Pakhtunkhwa, Pakistan; 4 Department of Biosciences, COMSATS University Islamabad, Islamabad, Pakistan; 5 Department of Biotechnology and Genetics Engineering, Kohat University of Science and Technology, Kohat, Khyber Pakhtunkhwa, Pakistan; 6 Department of Zoology, Hazara University, Mansehra, Khyber Pakhtunkhwa, Pakistan; Institute for Biological Research "S. Stanković", University of Belgrade, SERBIA

## Abstract

Cystic echinococcosis (CE) is a zoonotic helminthiasis caused by different species of the genus *Echinococcus*, and is a major economic and public health concern worldwide. Synthetic anthelmintics are most commonly used to control CE, however, prolonged use of these drugs may result in many adverse effects. This study aims to discuss the *in vitro/in vivo* scolicidal efficacy of different medicinal plants and their components used against *Echinococcus granulosus*. Google Scholar, ScienceDirect, PubMed and Scopus were used to retrieve the published literature from 2000–2020. A total of 62 published articles met the eligibility criteria and were reviewed. A total of 52 plant species belonging to 22 families have been reported to be evaluated as scolicidal agents against *E*. *granulosus* worldwide. Most extensively used medicinal plants against *E*. *granulosus* belong to the family Lamiaceae (25.0%) followed by Apiaceae (11.3%). Among various plant parts, leaves (36.0%) were most commonly used. Essential oils of *Zataria multiflora* and *Ferula asafetida* at a concentration of 0.02, and 0.06 mg/ml showed 100% *in vitro* scolicidal activity after 10 min post application, respectively. *Z*. *multiflora* also depicted high *in vivo* efficacy by decreasing weight and size while also causing extensive damage to the germinal layer of the cysts. Plant-based compounds like berberine, thymol, and thymoquinone have shown high efficacy against *E*. *granulosus*. These plant species and compounds could be potentially used for the development of an effective drug against *E*. *granulosus*, if further investigated for *in vivo* efficacy, toxicity, and mechanism of drug action in future research.

## Introduction

Most helminth parasites are broadly categorized into two main phyla, namely, nematodes (roundworms) including intestinal and filarial worms, and the Platyhelminthes (flatworms) including the flukes (trematodes) and tapeworms (cestodes) [1]. Helminthic parasite infections receive less than one percent of global research funding and therefore, are considered as neglected tropical diseases [2]. About 1/3 of the 3 billion people in the developing regions of the Americas, sub-Saharan Africa, and Asia living in low socioeconomic conditions are infected with one or more helminths [1].

Cystic echinococcosis is a zoonotic disease caused by the larval stages of the taeniid helminth *Echinococcus granulosus* [3] and is still a major economic and public health concern in several countries around the world [4]. CE is characterized by the long-lasting growth of hydatid cysts in the viscera of intermediate hosts such as sheep, cattle, goats, and humans [3], and can pose a serious health threat to humans depending upon the stage and location of the cyst. Usually, *E*. *granulosus* causes infection by forming cysts in the lungs, liver, brain or other vital organs [5]. CE is especially predominant in sheep and cattle raising regions of the world, including South and Central America, the Middle East, and the Mediterranean [6]. CE causes financial losses to the livestock industry in the form of condemnation of the infested meat [7], increased mortality, and weight loss as well as decreased milk production, decreased hide value and fecundity [8]. In addition, CE also results in morbidity and mortality in humans [4].

Treatment of the disease depends on stage, size, location, and complications of the cysts. At present, four treatment modalities are in practice for CE: surgery (the only treatment until the 1980s), chemotherapy with synthetic drugs like benzimidazole compounds, puncture aspiration injection and re-aspiration (PAIR), and the watch and wait method for clinically silent and inactive cysts [9]. However, these treatment methods have significant limitations. Some of the chemotherapeutic drugs used against CE are only used for inoperable cysts, though 20–40% cases do not respond favorably to those drugs and there are many related adverse effects [10]. During surgical practices, there is a high risk of intraoperative release of cystic fluids that subsequently results in secondary infection and relapse of hydatid cysts in approximately 10% of the cases [4]. To minimize the risk of recurrence the use of active scolicidal agents is indispensable [10]. Recently, it has been shown that existing scolicidal agents like cetrimide, ethanol, hypertonic saline, silver nitrate and others are related to severe side effects such as sclerosing cholangitis [11]. In traditional and rural settings, natural compounds from medicinal plants are being used as a remedy against CE because they are easily available and are thought to be efficacious while presenting fewer adverse side effects [12]. There are indeed a large number of medicinal plants whose scolicidal activity has been demonstrated against the protoscoleces of *E*. *granulosus*, however, there are many more plants which have not been explored yet.

We gathered the published literature on plants with anthelmintic/scolicidal activity against protoscoleces of *E*. *granulosus*. The purpose of this review is to better understand the current trends in research addressing the development of new scolicidal agents from plant sources. The findings of this review could help to provide up to date knowledge concerning scolicidal potential of medicinal plants and to exploit existing knowledge gaps to improve future research by recognizing areas where more focus should be given.

## Methodology

### Study design

This systematic review was designed and conducted according to the provided guidelines of Preferred Reporting Items for Systematic Review and Meta-Analysis (PRISMA) [13]. There is no specific protocol for conducting this systematic review. The PRISMA check list S1 Table is provided in the supporting information section.

### Search strategy

The search engines used for retrieving published data (from 2000 to 2020) include universally recognized databases, specifically, Scopus, PubMed, ScienceDirect, and Google Scholar. The search strategy was to download and retrieve published literature dealing with medicinal plants and compounds having scolicidal activity against *E*. *granulosus*. Specific keywords such as “scolicidal agents”, “medicinal plants used against *E*. *granulosus*”, “scolicidal activities of plant based compounds”, “*in vitro* or *in vivo* activity of plants against echinococcosis”, “natural products against protoscoleces”, “Natural scolicidal AND protoscolicidal”, and “scolicidal AND antihydatid NOT synthetic” were used.

### Inclusion/Exclusion criteria

Studies reporting *in vitro/in vivo* scolicidal efficacy of medicinal plants against protoscoleces of *E*. *granulosus* were included in this review. Moreover, studies reporting scolicidal activity of plant based pure compounds and studies with available full text were also considered for the current review. Studies reporting *in vitro/in vivo* anthelmintic activity of helminth parasites other than *E*. *granulosus*, studies concerning synthetic scolicidal agents against *E*. *granulosus*, studies concerning nanoparticles and invertebrates as scolicidal agents, epidemiological and molecular studies, and studies published in languages other than English were excluded.

### Study selection

Endnote (Thomson Reuters, San Francisco, CA, USA) was used to compile the articles. Initially, two investigators (RA and MR) assessed titles and abstracts of the retrieved articles for eligibility criteria. Then, the relevant full text published articles were reviewed by three investigators (RA, MR, and MK). In case of any controversy a fourth investigator (MA) was invited to discuss the article. Information including species of plant used, habitat, part(s) used, compound(s) used, concentration/dose, exposure time, scolicidal efficacy, and the name of the country in which the experimental work was performed was considered in the selection process. “Plant list” (http://www.theplantlist.org) and “Tropicos” (http://www.tropicos.org) were referenced for the standardization of plant scientific names, synonyms and families. PubChem (https://pubchem.ncbi.nlm.nih.gov) was also used to attain the IUPAC name (s) of pure compounds isolated from different plants. The summary measures were descriptive.

The software “MarvinSketch (18.24.0)” and “Inkscape (0.92)” were used to draw chemical structures of the compounds and figures/ illustrations, respectively.

## Results

**Fig 1:**
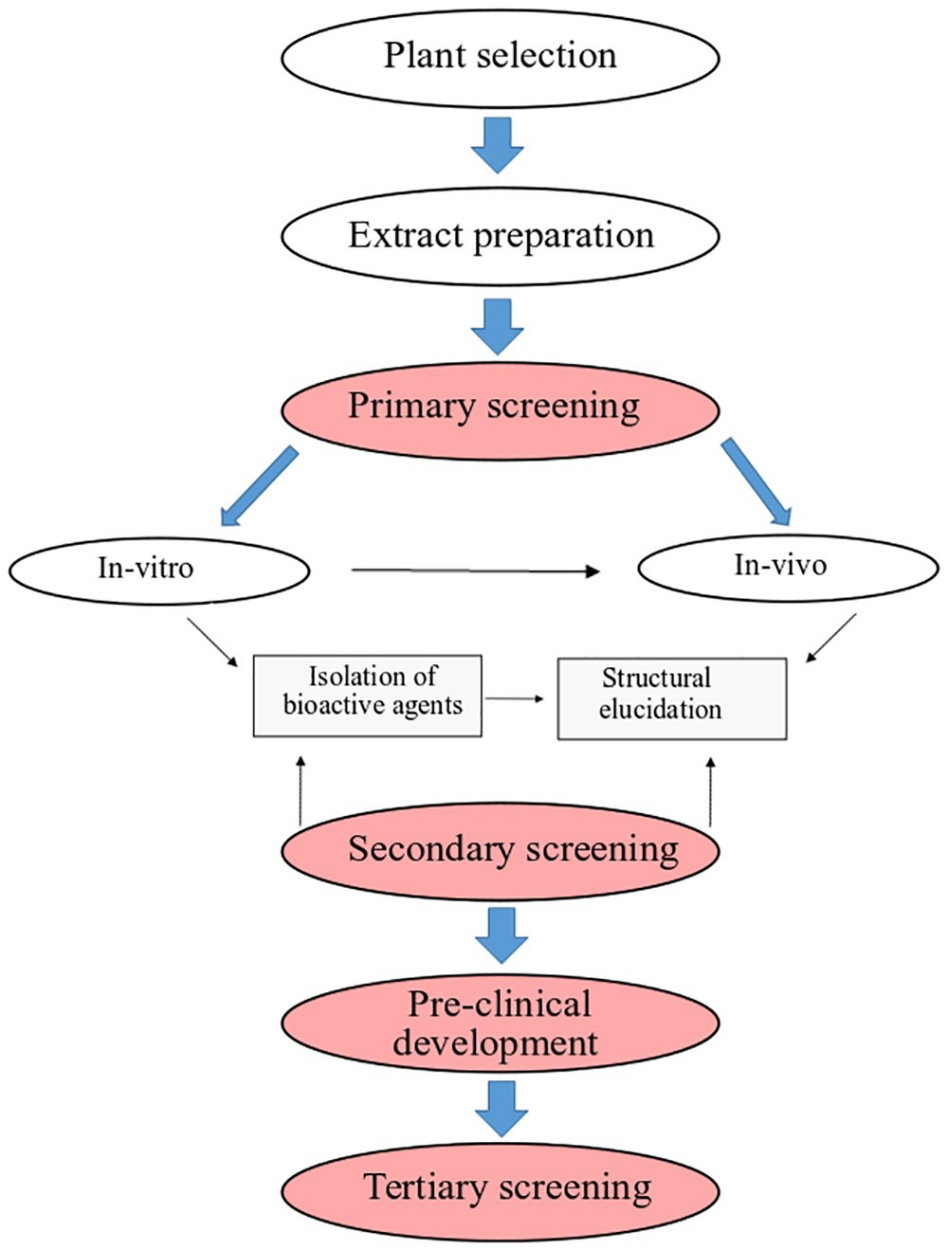
Strategy for drug development from medicinal plants.

**Fig 2:**
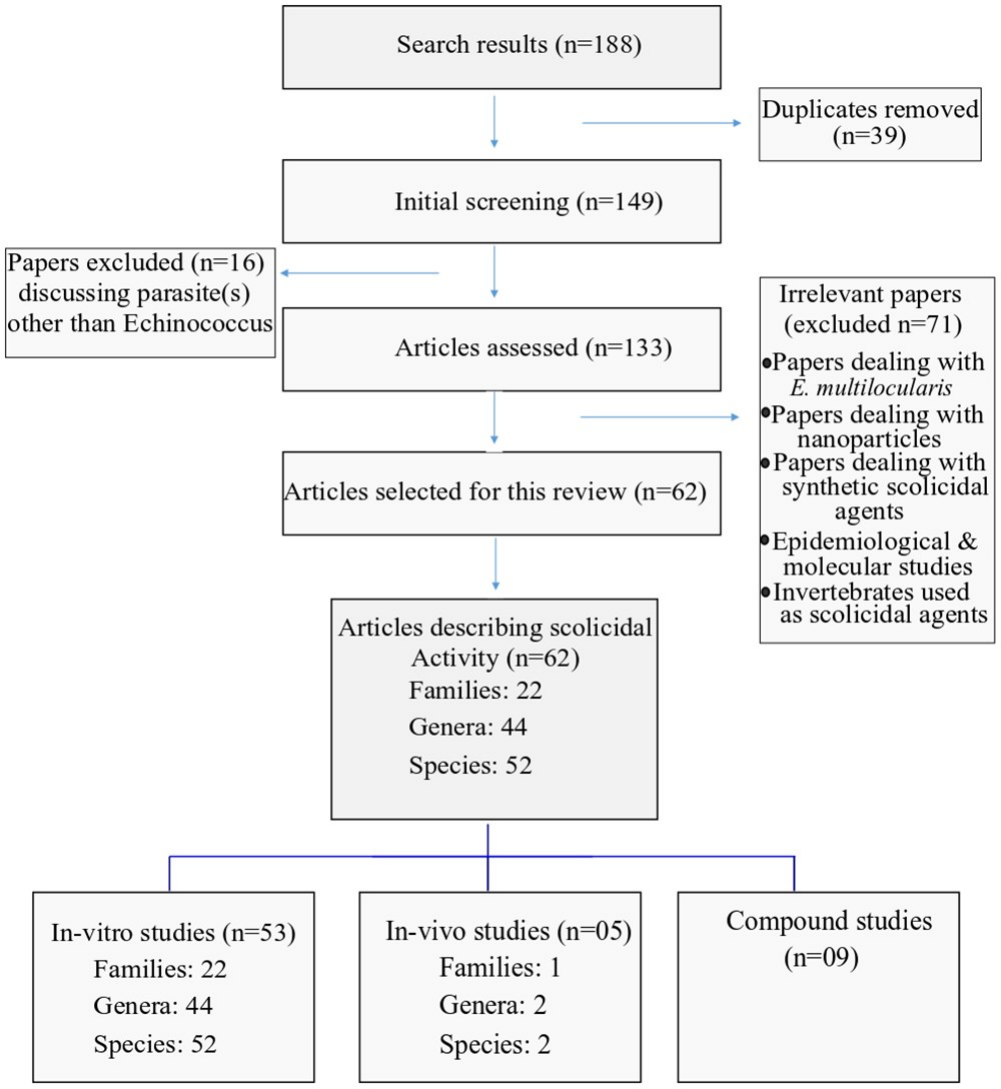
Flow chart of screening process for this review.

**Fig 3:**
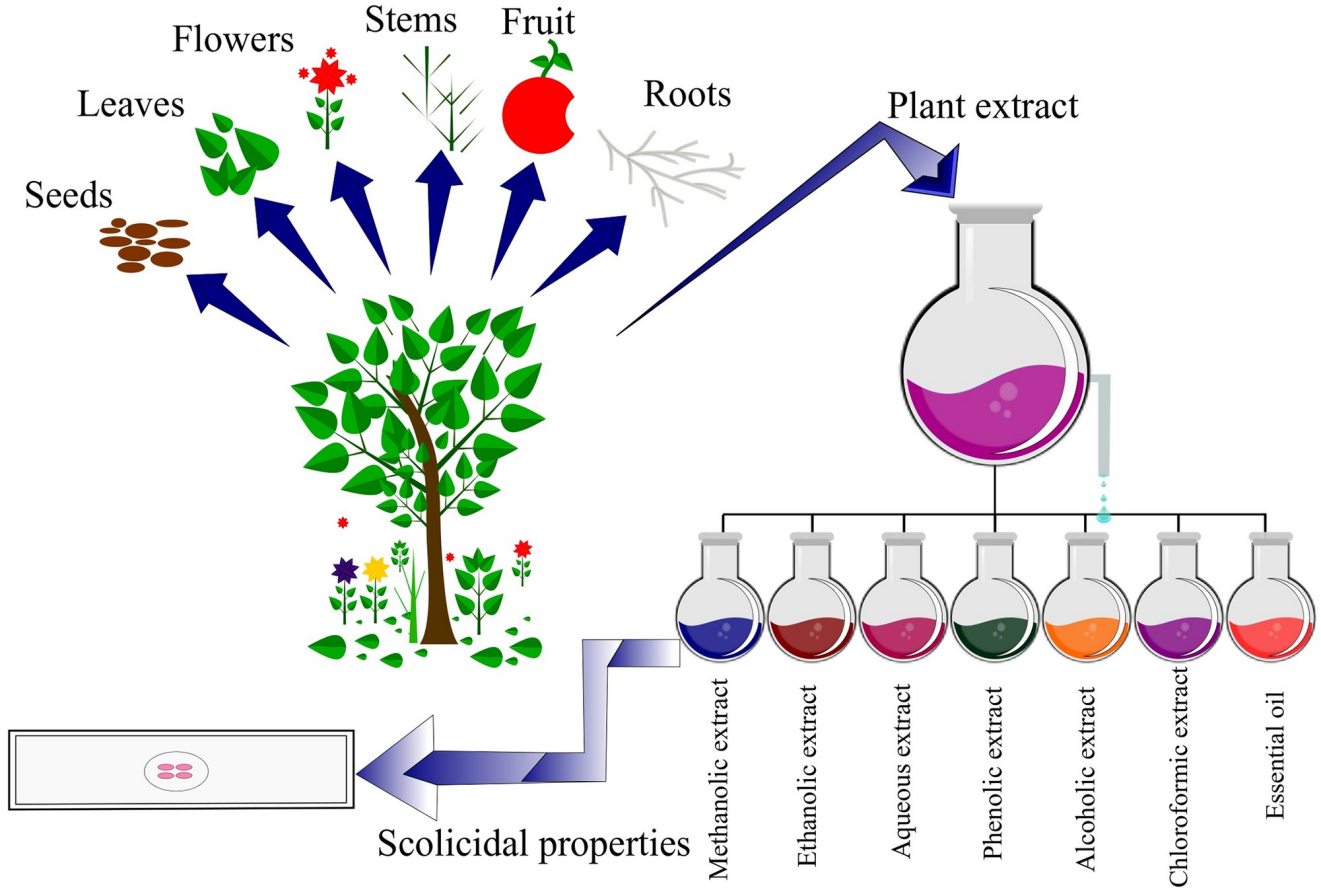
Schematic representation of medicinal plants and their extracts of various parts used against protoscoleces of *E*. *granulosus*.

Medicinal plants with reliable therapeutic effects are valuable for modern systems of herbal and natural drug discovery. Plants could serve as a direct source of bioactive or therapeutic agents, and these bioactive ingredients act as a raw material for the development of more complex semisynthetic chemical compounds. Isolated compounds of medicinal plants can lead to the discovery of new drugs, and finally, plants can be used as bioactive markers for spectroscopic and chromatographic analyses along with the discovery of new compounds [14] **(**Fig 1). This review was designed to discuss those medicinal plants and their compounds which have proven scolicidal activity against the protoscoleces of *E*. *granulosus*. We identified a total of 188 published articles through literature search. After removing duplicates and irrelevant articles, a total of 62 articles were selected for this review. Among the 62 studies, 53 studies evaluated *in vitro* activity of medicinal plants, while 5 studies evaluated *in vivo* activity, and 9 studies discussed plant compound activity against *E*. *granulosus* (Figs 2 and 3).

### Scolicidal medicinal plants, their families, and habitat

**Fig 4:**
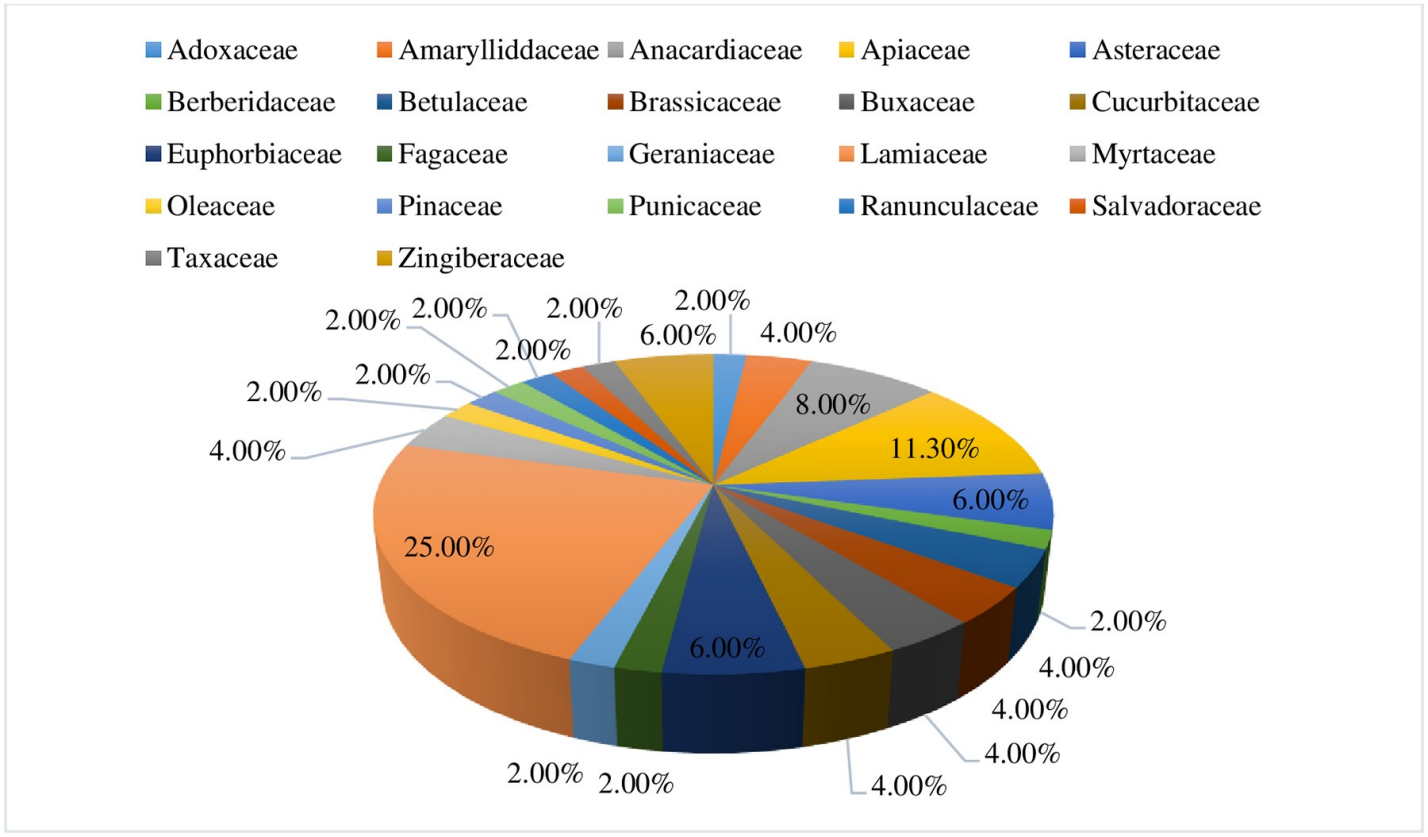
Percentage of plant families evaluated for scolicidal activity.

We reported a total of 52 plant species, belonging to 44 genera and 22 families, which have pharmacological validation and scolicidal activity against protoscoleces of *E*. *granulosus*. Plant families including Lamiaceae (n = 13, 25.0%), Apiaceae (n = 5, 11.3%), Anacardiaceae, Myrtaceae (n = 4, 8.0% each), and Euphorbiaceae (n = 3, 6.0%) were among the most commonly used plant families **(**Fig 4). The plant family Lamiaceae, Apiaceae, Anacardiaceae, and Myrtaceae were predominantly used in Iran, while Euphorbiaceae was reported to be used in India and Yemen. Herbs (n = 27, 61.4%) were most often used followed by trees (n = 11, 25.0%), and shrubs (n = 9, 28.0%).

Most of the studies describing scolicidal activities of medicinal plants were reported to be carried out in Iran (n = 43, 63.3%), Egypt (n = 4, 6.3%), and Argentina (n = 3, 5.0%). Tow studies each were carried out in India, Iraq, Saudi Arabia and Yamen, and the countries of Algeria, Pakistan, China, Turkey, and Switzerland each reported only a single study.

### Parts used and herbal formulation of medicinal plants used as scolicidal agent

Leaves were found to be the most frequently used, being the part used in 36.0% of the studies, followed by seeds (16.0%), fruit (14.3%), aerial parts (7.1%), and roots (5.3%). Other parts used included cloves, flowers, stems, whole plants, and latex. Results of this review revealed that essential oil was the preferred method of herbal formulation was used in 28 different recipes, while methanolic extract (in 23 recipes) was the second most used herbal formulation (Table 1). All formulations were made from only a single species of the plant, and no polyherbal formulation was reported in this review against protoscoleces of *E*. *granulosus*.

**Table 1:** *In vitro* scolicidal efficacy of various medicinal plants and their parts used against protoscoleces of *E*. *granulosus*.

Plant		Part used/ Location	Extract	Major phytochemical components (%)	Minimum concentration (mg/ml)	Exposure Time (min)	Maximum scolicidal efficacy (%)	Ethnomedicinal/ pharmacological uses	Citations
Family	Botanical name/ Common name/Habit								
Adoxaceae	*Sambucus ebulus* L. (= *Sambucus humilis* Mill.)/ Elderberry/ Herb	Fruit/ Iran	Methanolic	Flavonoids, steroids, tannins, caffeic acid, ebulitins, *α*-triterpenes	100	60	98.6	Anti-inflammatory, anti-nociceptive, anti-cancer, anti-angiogenic, anti-oxidative	[15]
Amaryllidaceae	*Allium sativum* L./ Garlic/ Bulb	Cloves/ Iran	Aqueous	N/A	200	60	42.3	Antiviral, antibacterial, antifungal, antitumor, antioxidant, antihelminthic, antiprotozoal	[16]
			Hydro-alcohol			60	71.4		
			Chloroform			30	99.5		
			Methanolic	Alkaloids (2.56), saponin (4.60), flavonoids (1.16), steroids (0.04), cardenolides (0.20)	50	10	100		[17]
			Chloroformic	N/A	50	60	98		[18]
			Hydro-alcoholic			60	92		
		Flowers/ Iran	Ultrasonic	N/A	100	180	98		[19]
	*Allium cepa* L. (= *Allium angolense* Baker)/ N/A/ Bulb	Root/ Iran	Methanolic	Tannins, flavonoids, alkaloids	100	60	21.8	Anti-bacterial, anti-parasitic, anti-microbial, anti-ascorbic	[20]
Anacardiaceae	*Pistacia atlantica* Desf. (= *Pistacia mutica* Fisch. & C.A.Mey.) / N/A/ Tree	Fruit/ Iran	Methanolic	*β*-myrcene (41.4%), *α*-pinene (32.48%), limonene (4.66%)	50	10	100	Anti-inflammatory, Anti-oxidant, anti-tumor, anti-asthmatic, anti-microbial	[21]
	*Pistacia khinjuk*/ N/A/ Tree	Leaves/ Iran	Essential oil	Spathulenol (20.87), Germacrene B (9.53), *β*-pinene (1.49), Myrcene (2.85), α-pinene (2.11)	0.512	N/A	Strong	Gastralgia, dyspepsia, peptic ulcer, anti-inflammatory, antipyretic, antibacterial, antiviral	[22]
			Ethyl acetate				Weak		
			Ethyl alcohol				Weak		
			Chloroform	N/A			Weak		
	*Pistacia vera* L./ N/A/ Tree	Branch/stems/ Iran	Essential oil	Limonene (26.21), *α*-Pinene (18.07), *α*-Thujene (9.31), *α*-Terpinolene (9.28), Camphene (4.41), β-Pinene (3.06),	200	5	100	Anti-bacterial, anti-viral, anti-fungal, anti-parasitic, anti-inflammatory, anti-nociceptive, anti-athero-genic, anti-diabetic	[23]
	*Rhus coriaria* L. (= *Toxicodendron coriaria* (L.) Kuntze)/ Sumac/ Shrub	Fruit/ Iran	Methanolic	Tannins, flavonoids, terpenoids	50	10	100	Anti-oxidant, anti-fibrogenic, anti-bacterial, anti-diabetic, anti-tumorigenic, hypoglycemic	[24]
Apiaceae (= Umbelliferae)	*Bunium persicum* (Boiss.) B.Fedtsch (= *Carum persicum* Boiss.) / Zireh Siah/ Herb	Seeds/ Iran	Essential oil	γ-Terpinene (46.1), cuminaldehyde (15.5), cuminyl alcohol (7.4), *ρ*-Cymene (6.7), β-Caryophyllene (0.2)	25	5	100	Anti-spasmodic, antimicrobial, antioxidant, anti-inflammatory	[25]
	*Ferula assafoetida* L. (= *Ferula foetida* St.-Lag.) / Angozeh/ Herb	Latex/ Iran	Essential oil	α-Pinene (0.6), β-Myrcene (0.6), Decane (0.6)	0.06	10	100	Analgesic, anthelminitic, antiseptic, sedative, expectorant	[26]
		Latex/ India	Methanolic	Terpenoids, sulphide derivatives, phenols and minerals	30	60	93.70	Anthelmintic, antibiotic, antimicrobial, antifungal, anticancer, anti-diabetic and therapeutic properties	[27]
	*Ferula gummosa* Boiss./ N/A/ Herb	Leaves/ Iran	Essential oil	N/A	50ug/ml	60	100	N/A	[28]
	*Foeniculum vulgare* Mill. (= *Anethum dulce* DC.) / Fennel/ Herb	Seeds/ Iran	Essential oil	trans-anethole (36), α-pinene (20), limonene (13), methyle chavicol (88)	1	5	100	Anti-oxidant, cytotoxic, anti-inflammatory, anti-microbial, anti-mutagenic, anti-thrombotic, diuretic	[29]
	*Trachyspermum ammi* (L.) Sprague (*Ammi copticum* L.)/ Ajowan/ Herb	Fruit/ Iran	Essential oil	Thymol (50.07), γ-Terpinene (23.92), *ρ*-Cymene (22.9), Linalool (0.01)	5	60	100	Anthelminthic, insecticidal	[30]
		Latex/ India	Methanolic	Thymol, γ-terpinene, p-cymene	20	60	93.69	Anthelmintic, insecticidal and antiseptic properties	[27]
	*Coriandrum sativum* L./ N/A/ Herb	Seeds/ Iran	Phenolic	N/A	750	4,320	100	Anti-bacterial, anti-parasitic, anti-fungal	[31]
Asteraceae (= Compositae)	*Artemisia aucheri* Boiss./ Artemisia/ Herb	Fruit/ Iran	Methanolic	Linalool (27.1), Borneol (7.8), Decane (5.4), Lavandulol (4.1)	100	60	17.4	Anti-bacterial, ant-ileishmanial, anti-parasitic, antioxidant	[32]
	*Artemisia sieberi* Besser/N/A/ Herb	N/A/ Iran	Aqueous	N/A	75	10	92.6	spasmolytic, wormicide, anti-inflammatory, anti-oxidant, antifungal, antimicrobial, anti-tumors	[33]
	*Tripleurospermum disciforme* (C.A.Mey.) Sch.Bip./N/A/ Herb	Leaves/ Iran	Methanolic	N/A	50	10	100	Anti-inflammatory, anti-spasmodic, Anti-septic, antibacterial,	[34]
Berberidaceae	*Berberis vulgaris* L. (= *Berberis abortiva* P.Renault)/ Barberry, Zark/ Shrub	Fruit/ Iran	Aqueous	N/A	4	5	100	Anti-bacterial, anti-parasitic, anti-fungal	[35]
			Hydro-alcoholic		2				
		Root/ Iran	Methanolic	Isoquinoline alkaloid, carotenoid, flavonoid, tannin, flavonol, triterpene	2	10	100		[36]
		Arial parts/ Pakistan	Ethanolic	N/A	50	50	65	Antimicrobial, antipyretic, antipruritic, antimetic and cholagogue actions, jaundice, dysentery, choleocystitis, leishmaniasis, gall stones and choleothiasis	[37]
Betulaceae	*Corylus* spp/ Hazel	Seeds/ Iran	Chloroformic	N/A	50	60	36	N/A	[18]
			Hydro-alcoholic			60	33		
Brassicaceae	*Cardaria draba* (L.) Desv. (= *Lepidium draba* L.) / Whitetop/ hoary cress/ Herb	Leaves/ Iran	Ethanolic	Isorhamnetin (13.8), Quercetin (12.9), Caffeic acid (7.2), Sinapic acid (6.7), Vanillin (6.4)	10	60	67.6	Anti-oxidant, anti-inflammatory, anti-parassitic, anti-bacterial	[38]
		Seeds/ Iran	Ethanolic	Caffeic acid (13.3), Sinapic acid (7.9), Quercetin (7.8), Vanillin (6.7), Isorhamnetin (6.4)	10	60	66.3		[38]
	*Lepidium sativum* L. (= *Cardamon sativum* (L.) Fourr.)/ Garden cress/ Herb	N/A	Essential oil	Thujene (88.86%), Myrcene (2.9%), *ρ*-cymene (1.67%)	10	30	100	Dysentery, diarrhea, skin diseases, diuretic, leprosy, asthma	[39]
Buxaceae	*Buxus wallichiana* Baill.*/* Shamshad/ Shrub	Arial parts/ Pakistan	Ethanolic	N/A	50	50	69.07	Bittertonic, diaphoretic, vermifuge, antihelmentic, antireumatic, analgesic, antiepileptic, antileprotic and in hemorrhoids	[37]
Cucurbitaceae	*Cucurbita* spp (= Cucurbits)/ N/A	Edible part/ Iran	Chloroformic	N/A	50	60	47	N/A	[18]
			Hydro-alcoholic			60	44		
	*Dendrosicyos socotrana* Balf. f./ N/A/ Tree	Leaves/ Yemen	Methanolic	Triterpenoids	5	21,600	100	Anti-malarial, anti-viral, urinary retention, cystitis, symptoms of diabetes, problems with the liver and burns, constipation	[40]
			Aqueous			24, 480	100		
Euphorbiaceae	*Euphorbia heliscopia/* Gandi Booti/ Herb	Arial parts/ Pakistan	Ethanolic	N/A	50	50	62.24	Edema, ascites, pulmonary tuberculosis, tinea, febrifuge, cathoratic, antihelminthic and purgative	[37]
	*Jatropha unicostata* Balf. f./ N/A/ Tree	Leaves/ Yemen	Methanolic	Flavonoids, terpenoids, fatty acids	5	17,280	100	Anti-viral activity against influenza type A, Herpes simplex 1	[40]
			Aqueous						
	*Mallotus philippinensis*/ Shaandendri/ Tree	Fruit/ India	Methanolic	N/A	2	60	99.2	Anti-bacterial, anti-retroviral, anti-viral, anti-oxidant, anti-inflammatory, anti-parasitic	[41]
Fagaceae	*Quercus brantii* Lindl. (= *Qurecus persica* Jaub. & Spach)*/* Oak/ Tree	Stem/ Iraq	Aqueous	Tannins, flavonoids, and phenolic	5	15,840	100		[42]
					10	14,400	100		
					15	12,960	100		
			Alakloid		5	14,400	100		
					10	12,960	100		
					15	11,520	100		
			Phenolic		5	8,640	100		
					10	8,640	100		
					15	7,200	100		
Geraniaceae	*Pelargonium roseum/*N/A/Shrub	Leaves/ Iran	Essential oil	N/A	0.05	60	100	Anti-trichomonal and insect Repellence	[28]
Lamiaceae	*Hymenocarter longiflorus*/ N/A/ Herb	Leaves/ Iran	Essential oil	α-Terpinene (0.11), Linalool (2.98), *ρ*-Cymene (0.2), trans-Caryophyllene (2.29)	N/A	N/A	Methanolic and essential oil extract showed significant scolicidal efficacy against *E granulosus* with LC50 values of 135.88 and 79.68 μm/ml respectively.	Antimicrobial, anti-mosquito agent, larvicidal	[22]
		Stems, inflorescences/ Iran	Methanolic						
	*Mentha piperita* L. (= *Mentha × adspersa* Moench)/ Peppermint/ Herb	Leaves/ Argentina	Essential oil	N/A	0.01	34,560	50	Anti-microbial, anti-viral, anti-oxidant, anti-allergic, anti-tumor	[43]
		N/A/ Argentina	Essential oil	N/A	0.01	10,080	77		[44]
	*Mentha pulegium* L. (= *Calamintha fenzlii* Vis.)/ Pennyroyal/ Herb	Leaves/ Argentina	Essential oil	*α*-Pinene (24.7), Linalool (12.6), Myrtenyl acetate (8.3), *α*-Terpineol (6.1), Linalyl Acetate (5.9), *α*-Terpinyl acetate (3.8)	0.01	25,920	100	Anti-inflammatory, antioxidant, anti-nociceptive, neuroprotective, anti-hepatic ischemia, anti-microbial	[43]
		N/A/ Argentina	Essential oil		0.01	10,080	82		[44]
	*Ocimum bacilicum*/ Sweet basil/ Herb	Leaves/ Iran	Methanolic	N/A	100	60	24.1	Anti-bacterial, anti-fungal and in treatment of splenomegaly	[20]
	*Origanum vulgare* L. (= *Origanum albiflorum* K.Koch)/ Oregano/ Herb	Leaves/ Argentina	Essential oil	Thymol (19,71), Carvacrol (5.4), γ-Terpinene (12.77)	N/A	86,400	23.5	Anti-bacterial, Anti-parasitic, anti-fungal	[45]
	*Salvia rosmarinus* Spenn. (*= Rosmarinus officinalis* L.)/ Rosemary/ Herb	N/A/ Argentina	Essential oil	Diterpenes, triterpenes, flavonoids,	0.01	10,080	71	Anti-oxidant, anti-inflammatory, anti-proliferative, anti-cancer	[44]
	*Salvia officinalis* L. (= *Salvia cretica* L.)/ Sage/ Shrub	Aerial parts/ Egypt	Alcoholic	Apigenin-7-O-glucoside, Apigenin-acetylglucoside, sorhamnetin-luteolin, Apigenin	2.5	8,640	100	Anti-bacterial, anti-microbial, anti-oxidant	[46]
	*Satureja hortensis* L./N//A/ Herb	Arial part/ Iran	Essential oil	N/A	2	20	100	Antimicrobial, Antibacterial Antioxidant, and antifungal	[47]
	*Satureja khuzestanica*/ Jamzad/ Herb	Leaves/ Iran	Ethanolic	Thymol (t), Carvacrol (94.9), Thymol acetate (t), γ-Terpinene (0.49), *ρ*-Cymene (0.55), α –Terpinen (0.26)	1	30	100	Antidiarrheal, antispasmodic, anti-inflammatory, vasodilator, antioxidant, antibacterial, antiviral, antifungal	[48]
		Leaves/ Iran	Essential oil	N/A	10	10	100		[49]
		Flowers/ Iran							
	*Thymus vulgaris* L. (= *Origanum thymus* Kuntze)/ Thyme/ Shrub	Leaves/ Argentina	Essential oil	N/A	N/A	86,400	38.1	Anti-microbial, anti-inflammatory, anti-amoebic, anti-fungal, anti-parasitic, anti-bacterial	[45]
		Aerial parts/ Egypt	Alcoholic	Thymol (65.4), Carvacrol (5.4), Borneol (0.7), Borrnyl acetate (0.1)	N/A	103,680	100		[46]
	*Zataria multiflora* Boiss. (= *Zataria bracteata* Boiss.) / Avishan Sherazi/ Herb	Leaves/ Iran	Essential oil	Thymol (40.8), Carvacrol (27.8), β-Caryophyllene (2.0), Linalool (1.7), α-Terpinolene (1.3), *ρ*-Cymene (8.4), Thymol acetate (0.5)	0.02	10	100	Analgesic, antiseptic, antibacterial, antifungal, antiprotozoal, anti-inflammatory, antioxidant, immunostimulant, pain-relieving	[26]
					12.5	5	100		[50]
			Methanolic	N/A	25	1	100		[51]
					20	10	100		[50]
			Essential oil	N/A	2	10	100		[52]
			Essential oil	N/A	20	15	100		[53]
	*Ziziphora tenuior* L. (= *Faldermannia parviflora* Trautv.)/ N/A/ Herb	Aerial parts/ Iran	Total extract	Polegon (87), thymol (3.4), piperitenone (12.19), mentha-2-ethanol (31.5), carvacrol (10.5), menthone (46.4), neomenthol (78.4)	25	10	25 mg/ml concentration exhibited the highest scolicidal activity after 10 minutes of post-incubation. Considering the effect of different fractions Fof *Z*. *tenuior* against protoscoleces, the ethanolic fraction showed the highest effect followed by ethyl acetate, petroleum ether, and chloroform, respectively.	Antiseptic, antifungal, antibacterial, used in uterine diseases and dysentery	[54]
Myrtaceae	*Eucalyptus globulus* Labill. (= *Eucalyptus gigantea* Dehnh.)/ Eucalyptus/ Tree	Leaves/ Iran	Methanolic	Alkaloids, glycosides, terpenoids, steroids, flavonoids, tannins	50	40	100	Antiseptic, antimicrobial	[32]
	*Myrtus communis* L. (= *Myrtus acuta* Mill.)/ Myrtle/ Shrub	Leaves/ Iran	Essential oil	*α*-Pinene (24.7), 1,8- Cineole (19.6), Linalool (12.6), Myrtenyl acetate (8.3), *α*-Terpineol (6.1), Linalyl Acetate (5.9)	100	5	100	Anti-inflammatory, anti-nociceptive, anti-oxidant, anti-microbial	[55]
		Leaves/ Iran	Methanolic	N/A	50	10	100	Anti-fungal and Antibacteria	[34]
Oleaceae	*Olea europaea* L./ Olive/ Tree	Leaves/ Iran	Ethanolic	Flavonols, caffeic acid, gallic acid, oleuropein	1	120	96.7	Anti-oxidant, anti-allergic, anti-inflammatory, anti-microbial, anti-tumor, anti-hypersensitivity, anti-anthrogenic	[20]
Pinaceae	*Pinus nigra* Arn. subsp. *pallasiana* (Lamb.) Holmboe /N/A/ Tree	Fresh needle/ Turkey	Essential oil	N/A	50	60	100	wound healing, Hemorrhoids, diabetes, liver diseases, cold, bronchitis, stomachache, and fungal infections on the skin	[56]
Punicaceae	*Punica granatum* L. (= *Punica nana* L.)/ Pomegranate/ Shrub	Fruit peel/ Algeria	Aqueous	Triterpenoids, Steroids, Glycosides, Flavonoids, Tannins	16	66,240	100	Anti-coccidial, anthelmintic, anti-bacterial, anti-oxidant, anti-inflammatory, anti-microbial	[57]
Ranunculaceae	*Nigella sativa* L. (*Nigella cretica* Mill.)/ Black cumin seed/ Herb	Seeds/ Iran	Essential oil	Thymoquinone (42.4), *ρ*-Cymene (14.1), carvacrol (10.3), longifolene (6.1), 4-terpineol (5.1), t-anethole (2.3), limonene (1.7), thymol (1.2)	10	10	100	Anti-inflammatory, cough, bronchitis, eczema, influenza, anti-microbial, anti-cancer, anti-oxidant, anti-bacterial, anti-viral, anti-fungal, anti-parasitic	[58]
			Methanolic	N/A	50	10	100		[59]
			Aqueous			30	100		
		Seeds/Iraq	Aqueous	N/A	25	10,080	62.3		[60]
		Seed/ Egypt	Essential oil	N/A	100	120	100		[61]
Salvadoraceae	*Salvandora persica*/ Arak/ Shrub	Roots/ Saudi Arabia	Ethanolic	Alkaloids, tannins, saponins, flavonoids, sterols, terpenoids	50	20	100	Anti-inflammatory, analgesic, anti-microbial, anti-bacterial, anti-plaque	[62]
Taxaceae	*Taxus baccata* L./ N/A/ Tree	Gum resin/ Iran	Ethanolic	N/A	150	60	66.6	N/A	[63]
Zingiberaceae	*Curcuma longa* L. (= *Amomum curcuma* Jacq.)/ Turmeric/ Herb	N/A/ Saudi Arabia	Ethanolic	Alkaloids, saponins, flavonoids, terpenes, steroids	50	30	100	Anorexia, biliary disorders, cough, coryza, sinusitis, rheumatism, anti-inflammatory	[64]
	*Curcuma zadoaria* L./N/A/ Herb	Rhizome/ Iran	Aqueous	oxygenated monoterpenes, sesquiterpene hydrocarbons and oxygenated sesquiterpenes;	75	40	100	Hepatoprotective, anti-cancer, anti-analgesic, anti-allergen, and antimicrobial	[65]
	*Zingiber officinale* Roscoe (= *Amomum zingiber* L.)/ Ginger/ Herb	Rhizome/ Iran	Methanolic	Camphene (15.9), α–terpineol (8.8), farnesene (8.8), p-cineole (8.4), zingiberene (7.5), β-mycrene (7.7)	100	40	100	Arthritis, atherosclerosis, migraine headaches, rheumatoid arthritis, high cholesterol, ulcers, depression, impotence, common cold, antioxidant, antimicrobial, anti-inflammatory, antifungal	[32]
			Crude Methanol	N/A	25	60	100		[66]
		N/A/ Saudi Arabia	Ethanolic	N/A	50	10	100		[64]
		N/A/ Iran	Ethanolic	N/A	150	60	92.3		[67]

N/A indicates data not available

### *In vitro* scolicidal activities of medicinal plants

**Fig 5:**
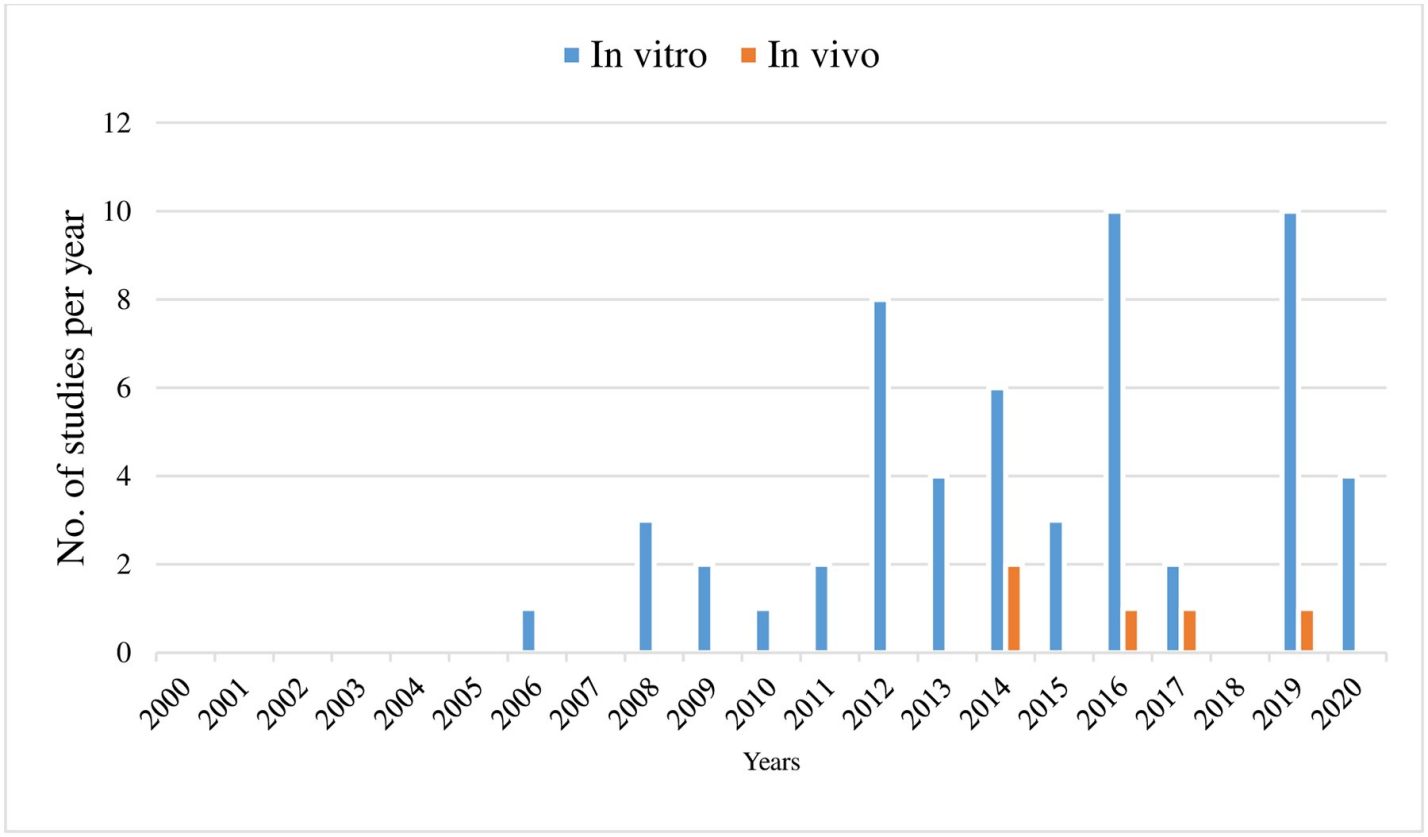
Year-wise comparison of *in vitro* and *in vivo* studies.

We reported a total of 52 plant species of (22 families), used *in vitro* against the protoscoleces of *E*. *granulosus* **(**Fig 5). Plant species (n = 13) of the family Lamiaceae were predominantly used *in vitro* against larvae of *E*. *granulosus*. Leaves among plant parts, herbs among plant life forms, and essential oil among extracts were dominant in *in vitro* studies (Table 1). Among the reported antihydatid medicinal plants, *Zataria multiflora* Boiss, *Ferula asafetida* L., and *Foeniculum vulgare* Mill. were found to be highly effective *in vitro*. Essential oil of *Z*. *multiflora* at a concentration of 0.02 mg/ml after exposure for 10 min caused 100% mortality of the protoscoleces. Similarly, *F*. *assafoetida* and *F*. *vulgare* essential oil at 0.06 and 1 mg/ml concentration were 100% effective after 10 and 5 min, respectively.

### *In vivo* scolicidal activities of medicinal plants

Several medicinal plants and pure compounds were reported that are being investigated for preventive and therapeutic activities against *E*. *granulosus* to underpin new alternative treatment for CE with fewer or less severe side effects. A total of 2 plant species, namely *T*. *vulgaris* and *Z*. *multiflora* of the family Lamiaceae (22 families reported against *E*. *granulosus* in this review) were potentially used in *in vivo* scientific validation of medicinal plants against the protoscoleces of *E*. *granulosus* **(**Fig 4). Extracts of leaves and whole plants were tested using animal models to validate the scolicidal efficacies *in vivo*. Mice (*Mus musculus*) were reported to be used as the animal model in *in vivo* studies (Table 2).

**Table 2:** *In vivo* scolicidal efficacy of medicinal plants against *E*. *granulosus*.

Plant		Part used/ location	Extract	Compound used	Concentration (mg/kg)	Time (days)	Animal model	Effect	Reference
Family	Botanical name/ common name								
Lamiaceae	*T*. *vulgaris*/Thyme/ Shrub	-/Argentina	Olive oil	Carvacrol*	40	20	Female CF-1 mice	Cysts were reduced in size and germinal layer of cysts lost their multicellular structure feature.	[68]
	*Z*. *multiflora* Boiss/ Avishan Sherazi/ Herb	Leaves/ Iran	Methanolic	-	4	243	Balb/C mice	Proved preventive and therapeutic efficacy. Moreover, weight and size of the cysts decreased and the germinal layer was completely damaged.	[69]
					8	30			
		Whole plant/ Iran	Nano emulsion Essential oil	-	20	60	Female mice (*Mus musculus*)	Recovered cysts were significantly reduced in size and number.	[52]
		Aerial parts/ Iran	Aromatic water	-	20000	243	Balb/C mice	Showed preventive and therapeutic effects and the germinal layer of hydatid cysts were completely damaged	[70]
					40000	30			
		Aerial parts/ Iran	Aromatic water	-	50	60	Female mice (*Mus musculus*	*Z*. *multiflora* AW in combination with Albendazole significantly reduce the size and weight of cysts.	[71]

Key: DMSO = dimethyl sulphoxide;

*purchased from Sigma-Aldrich

### Scolicidal activities of active phyto-compounds

A total of 8 active compounds, which were isolated from different medicinal plants, are documented in this review article. Of the reported active compounds, thymol, carvacrol, menthol, berberine, genistein, thymoquinone, ampelopsin, and gallic acid are included. All of these 7 compounds were investigated for *in vitro* efficacy against the protoscoleces, while only 2 compounds (thymol and carvacrol) were used in *in vivo* assays. Thymol, berberine, and thymoquinone revealed significant *in vitro* scolicidal activity at concentration of 0.1, 0.5, and 1 mg/ml after 5, 10, and 1 minute of exposure (Table 3). Thymol and carvacrol also showed promising scolicidal activity in *in vivo* assays (Table 2).

**Table 3:** Various plant-based compounds protoscolicidal efficacy used against *E*. *granulosus*.

Compound	IUPAC name	Chemical structure	Solvent used	Minimum concentration (mg/ml)	Time exposure (min)	Maximum efficacy (%)	Product company	Plant species	References
Thymol	2-isopropyl-5-methylphenol	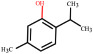	Dimethyl sulphoxide	0.01	115,200	100	Sigma Aldrich (USA)	*T*. *vulgaris*	[72]
			Dimethyl sulphoxide	0.005	10,080	63	Sigma Aldrich (USA)	*O*. *vulgare*	[44]
			Distilled water	0.05	8,640	100	BDH Chemicals LTD (Poole, UK)	*Z*. *multiflora*	[46]
			Normal saline plus Tween 80	0.1	5	100	Sigma-Aldrich (St. Louis, MO)		[73]
Menthol	2-Isopropyl-5-methylcyclohexanol	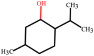	Distilled water	0.05	4,320	100	El-Nasr Company for Pharmaceuticals and Chemicals (Cairo, Egypt).	*T*. *vulgaris*	[46]
								*S*. *officinalis*	
Berberine		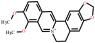	Dimethyl sulfoxide	0.5	10	100	Sigma-Aldrich, (St. Louis, MO, USA)	*B*. *vulgaris*	[36]
Thymoquinone	2-Isopropyl-5-methyl-1,4-benzoquinone	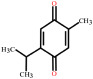	Dimethyl sulfoxide (DMSO)	1	1	100	Sigma-Aldrich (St. Louis, Missouri, USA)	*N*. *sativa*	[58]
Gallic acid	3,4,5- trihydroxybenzoic acid	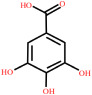	Distilled waster	35	3	100	Sigma-Aldrich (St. Louis, MO)		[74]
Carvacrol	5-Isopropyl-2-methylphenol	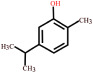	Normal saline plus Tween 20	100	5	100	Sigma-Aldrich (St. Louis, MO)	*Z*. *multiflora*	[73]
Genistein	4’,5,7-Trihydroxyisoflavone	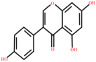	Dimethyl sulfoxide	0.01	5,760	60	Synthesized at the Department of Chemistry, University of Liverpool		[75]
Ampelopsin	(2R,3R)-3,5,7-trihydroxy-2-(3,4,5-trihydroxyphenyl)-2,3-dihydrochromen-4-one	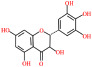	Dimethyl sulphoxide	160 μM	10080	100	-	*Ampelopsis grossedentata*	[76]

### Toxicity of scolicidal medicinal plants and compounds

Generally, toxicity tests are performed for the purpose to evaluate the toxicity levels of herbal formulations. Optimal scolicidal agents are those that are nontoxic and destroy the protoscoleces in short period of time and at minimum concentration [77]. Results of the review revealed that the only one plant species, *Z*. *multiflora*, has been evaluated for toxicity along with 2 compounds, viz. thymol and carvacrol. In a study, the extract of *Z*. *multiflora* was proven to be safe and have no toxic effects when used in pregnant “Blab/C mice” [69, 70]. Similarly, oral administration of 40 mg/kg of thymol and carvacrol revealed no toxic effects in the CF-1 mice during the course of the experiment [68, 78].

## Discussion

A total of 52 plant species were reported to be pharmacologically evaluated for their scolicidal activity against protoscoleces of *E*. *granulosus* in this review, most of which belong to the families Lamiaceae, Apiaceae, Anacardiaceae, Myrtaceae, and Euphorbiaceae. The traditional beliefs of herbalists, high abundance of these plant families, and the presence of phenolic compounds, essential oil, and saponins in Lamiaceae [79], polyacetylenes in Apiaceae [80], and terpenoids and alkaloids in Euphorbiaceae family [81] could be possible reasons behind such an extensive use and anthelmintic activity of these plant families. Plants of the family Lamiaceae could be easily cultivated and propagated. Moreover, they are extensively used for their strong aroma and ability to survive in severe hot weather because of their essential oils (122). The extensive use of the Euphorbiaceae family for various medicinal purposes may be attributed to its global distribution and mode of adaptation in dry conditions because of the succulent nature of its species and CAP pathway ability. Plant species of this family possess a wide array of secondary metabolites and tendency of mutation load due to their exposure to a wide range of environmental conditions (126). Herbs were reported to be the most frequently used form of plant life used against helminth parasites as compared to shrubs and trees. The dominancy of herbs over other forms of life could be attributed to their easy availability and high efficacy against different ailments as compared to shrubs and trees [82]. Tariq et al. (2017) reported that herbs are widely used in folk medicines all over the globe and contain a large number of active compounds which are responsible for their high efficacy and make them the first choice for scientists and traditional healers [83]. Our results showed that trees are the least commonly utilized plant life form when compared to herbs and shrub, which is possibly due to threats to biodiversity loss and ecosystem consequences. According to Sadia et al. (2018) several tree species have been placed under key protection due to over harvesting. In these situations, modern techniques i.e. cloning, callus cultivation, cultivation in nature, genetically transformed cultures and propagation should be used to obtain the chemical constituents of medicinal importance and to overcome the supply demand imbalance [84].

It was worth mentioning that most (68.3%) studies were conducted in Iran only and the remaining (32.0%) studies in other countries of the world including Argentina, Pakistan, Turkey, Saudi Arabia, Yemen, India, Iraq, Algeria, Egypt and Switzerland. The reason behind such an exceptional number of studies on control of CE in Iran could be the fact that agriculture is the main economic sector that contributes more than 25% of GDP and 1/3 of total employment in Iran. About 90% of the population food requirements are covered by domestic production and domestic supplies cover 95% of agro-industry needs [85]. The livestock sector plays an essential role in the livelihood of the rural population that is mainly dependent on livestock by providing about 80% of their income and on average, 31.8% of the gross value of agriculture production is attributed to livestock production. By-products of livestock e.g. hair, hides, intestines, milk, and red meat are among the major sources of the country’s economic activity. Sheep and goats solely produce about 53% of the red meat while the production of red meat and milk increased during the last decade by 3.14% and 7.19% annually [86]. Other countries such as Argentina, Pakistan, Turkey, Saudi Arabia, Yemen, India, Iraq, Algeria, Egypt and Switzerland contributed to the studies on pharmacological validation of medicinal plants against *E*. *granulosus* but their contributions are less in number. This supports the notion that CE is a highly underappreciated threat to human health along with other neglected helminthic infections all over the world. There is a dire need of research on CE and researchers are invited from all over the world to explore the existing knowledge gaps, as there are many medicinal plants and their compounds which are still unexplored that may prove useful in future research.

Among all the plants parts, leaves were most frequently reported to be used during pharmacological validation of medicinal plants against larvae of the helminth parasite *E*. *granulosus*. According to Moshi et al. (2012) leaves are preferred by herbalists because they prefer a sustainable supply of raw materials [87]. Moreover, leaves can be easily harvested without extensively harming plants and this could be the possible reason that leaves are the most utilized plant part [88]. Tariq et al. (2017) reported that leaves contain different bioactive compounds which cause a variety of medicinal effects [83]. In contrast, Albuquerque (2006) found that such an exceptional use of leaves in herbal medicines could possibly slow down the process of plant growth, which would lead to infrequent plant recipes [89]. Seeds of plants contain flavonoids, saponins, and tannins etc. and it seems that these phytochemical compounds play key roles in the bioactivity of medicinal plants [90]. Roots act as storing organs of various nutrients for plants, which could explain why they are extensively used in herbal medicines [91]. However, root collection usually results in the death of the plant and can pose serious threats to conservation [79]. Similarly, harvesting of whole plants for evaluation of their anti-parasitic activities is also problematic from a conservation point of view [92]. Extensive use of essential oil and methanolic extract emphasize the role of solvents in extraction of potential bioactive compounds from different plants and their parts as well. Methanol, due to its polar nature, is extremely effective in extracting bioactive compounds from plants [93], and this could be the possible reason of such an extensive of this solvent is herbal formulations. On the other hand, essential oils have proven anthelmintic activity [94], moreover they are comprised of terpenes (secondary metabolites) that interfere with biochemical and physiological functions of parasites. No polyherbal formulation was reported from the pharmacological validations of medicinal plants against protoscoleces of *E*. *granulosus*, even though combination of different plants and extracts are commonly more effective than sole plant/extract [95]. This is a clear gap and in future research polyherbal formulations should be evaluated to obtain promising results.

*In vitro* confirmation of the mentioned medicinal plants reveals the proof of reliability of the plants against the protoscoleces of *E*. *granulosus*. Lamiaceae family among plant families, leaves among plant parts, while herbs among plant life forms were predominant in *in vitro* evaluation of the medicinal plants against larvae of *E*. *granulosus*. *In vitro* studies mostly utilized essential oils for evaluation of scolicidal activity. This could be attributed to the fact that secondary metabolites present in the essential oils inhibit parasite’s biochemical and physiological functions and thus causing death of the cells [96, 97]. To obtained promising results, from a plant’s active compounds, are mainly depend upon the solvent used in the herbal formulation.

From the results it is evident that most of the studies are focused on *in vitro* rather than *in vivo* evaluation of plants against the protoscoleces of *E*. *granulosus*. This could be attributed to the fact that *in vitro* screening of plants is cost effective, less time consuming, and quick turnover of results, which allow plants screening on a large scale. In addition, these tests measured the effect of anthelmintic activity directly on the process of hatching, development, and motility of parasites without interfering the internal physiological functions of the hosts [98]. Another advantage of *in vitro* studies is that after getting reliable results, then the extract/compound could further be evaluated *in vivo* [99]. However, compounds/extracts that are effective *in vitro* may or may not active *in vivo* to the same extent [100]. This type of discrepancy in the activity of testing new anthelmintic drugs is relatively common which may be attributed to several factors such as (a) bioavailability and intrinsic pharmacology of the compound tested (b) the possible destruction or insolubility of the compounds in the rumen of animal (c) and additional protection of the parasite [101]. This limitation/gap signifies the importance of pharmacokinetics and pharmacodynamics studies for the industrial development of new anthelmintic products against *E*. *granulosus*.

Essential oils of *Z*. *multiflora* Boiss depicted promising antihydatid activity at the possible low concentration (0.02 mg/ml) and minimum time exposure, this potent activity of *Zataria* oil probably be due to the major phenolic monoterpenes components. Phenolic monoterpenes antimicrobial activity could be related to its intrinsic hydrophobicity and in addition the presence of a hydroxyl group; thus these compounds cause cells disruption by crossing the cell membrane [102, 103]. Mechanism of action of phenolic monoterpenes is not evaluated against protoscoleces yet, however, studies on other eukaryotic cells revealed that phenolic monoterpenoids mainly act on the plasma and mitochondrial membranes and induce cell apoptosis. They diffuse through membrane, disturb lipid bilayer structure, and hence change cell permeability, which in turn enhances the leakage of ions and reduces membrane electric potential. This disturbance in electric potential of plasma membrane eventually leads to the leakage of ATPs, amino acids, proteins, and ions especially potassium and calcium, which induce membrane damage and cell death [104–106]. Similarly, altering the biochemical structure of mitochondrial membrane results in the leakage of proteins, radicals, calcium, and cytochrome c, which cause cell death by apoptosis [107–109].

*F*. *assafoetida* and *F*. *vulgare* essential oil was relatively more effective in terms of efficacy and time exposure than other plants reported in this review. The essential oils from these plants bearing disulphide compounds, tested against various eukaryotic cancerous cells for their cytotoxic activity, which are also taught to be responsible for scolicidal activity [110, 111].

Mechanisms of drug action of many antihydatid plants are not evaluated and there is a dire need of research on this aspect to provide a detailed information on the scientific background of ethnomedicinal plants for the development of a novel scolicidal agent.

*In vivo* studies are desirable to assess the pharmacokinetics/pharmacodynamics of the target extract/compound, *in vivo* efficacy, host immune response to the target extract/compound, and toxicity levels. Besides a number of advantages of *in vivo* studies, there are also some disadvantages as well. *In vivo* studies would be more accurate and precise as compared to *in vitro*, but more timing consuming, costly, and difficult to reproduce due to the inter animal and pharmacodynamics in the host [112]. In short, both of the techniques have vital roles to play and the one will not exclude the other [113].

The two plant species *T*. *vulgaris* and *Z*. *multiflora* Boiss of the Lamiaceae family were used *in vivo* against protoscoleces. Use of the family Lamiaceae and plant part leaves was in accordance with ethnomedicinal use, where this family and leaves were extensively used. This shows the reliability and beliefs of modern science on ethnomedicines. Mouse is the most commonly used model for *in vivo* echinococcosis studies. The reason could be the high similarity of its genome with that of humans. Moreover, its small size, short generation time, and easy to breed, makes it an efficient cost effective model for *in vivo* studies and to get functional information about the human health and diseases [114, 115].

We reported a total of 8 compounds, which were isolated and evaluated for scolicidal activity against the protoscoleces. Plants have biologically active compounds in the form of primary and secondary metabolites. Among these, chlorophyll, proteins, and sugars are included in primary, while flavonoids, alkaloids, terpenoids, and phenols are the secondary compounds [116]. These different bio-active compounds work synergistically in combination to produce a pharmacological effect [117]. The efficacy of plant compounds may be attributed to the fact that they inhibit or retard the growth, maturation damage, suppress appetite or reduce procreative ability, which are all the causes of mortality. Moreover, the considerable activity of plants extracts may be due to the additive or synergistic relationship among different major components which can interact with multiple molecular targets in various developmental stages of the parasite [118]. Thymol, berberine, and thymoquinone revealed significant *in vitro* scolicidal activity at concentration of 0.1, 0.5, and 1 mg/ml after 5, 10, and 1 minute of exposure [36, 72]. Thymol effects were promising that could be explained by the fact that, it induces shrinkage of the soma region, loss of turgidity, rostellar disorganization, hooks loss, formation of blebs on the tegument, and destruction of microtriches, which finally lead to death of the protoscoleces [72]. This could be attributed to the fact that any damage to hooks and blebs formation on the tegument of protoscoleces are generally stress responses which are brought about by different harmful conditions. Moreover, microtriches destruction could affect the absorption of nutrients in protoscoleces and cause starvation and finally death of the larvae [119].

Berberine is an alkaloid broadly used in ethnomedicinal systems of Ayurveda and Traditional Chinese Medicines. Berberine is reported to inhibit growth and induce morphological changes which could be the possible reason of the mortality of the parasites [120]. Thymoquinone is the main component of essential oil of *Nigella sativa*, the exact mechanism of its effects in not evident, however, studies suggest that it can inhibit DNA synthesis by inhibiting histone deacetylase (HDAC) enzyme interacting with the chromosomes [121].

Thymol at concentration of 40 mg/kg showed both therapeutic and preventive effects. Ultrastructural observation revealed that the germinal layer was highly damaged and this could be attributed to the fact that the drug enters the hydatid cyst to cause the effect [122].

Moreover, studies concerning isolation and purification of plant compounds responsible for anti-parasitic activities are very scarce and insufficient. Most of the studies reported the presence of key components of the plants which do not provide information about the effective anti-parasitic compounds and their mechanism. Therefore, more in depth studies are required to evaluate mostly used plants of the aforementioned plant families.

Safety issues of herbal medicines have been remained a big question and scientists are taking keen interests in herbal medicines for a decade. The notion that ‘natural’ equals ‘safe’ is apparently deceptive, since natural products comprise pharmacologically active compounds which, when taken in high doses or in specific conditions, can be detrimental to health [83].

The *in vitro* and *in vivo* cytotoxicity of thymol was assessed by Robledo et al., 2005 [123]. The cytotoxic effect was observed to be 400 ± 0 μg/ml in U-937 human promonocytic cells. An oral dose of 40 mg/kg of body weight/day, thymol was not toxic to Golden hamsters based on corporal weight, behavior and serum levels of bilirubin, uric acid, and glucose [123]. Moreover, the toxicity evaluation of thymol in mice was also reported to safe and non-toxic, no changes in mice behavior, mobility, and feeding habits were observed [122]. Berberine at the tested clinical doses is not cytotoxic and mutagenic, whereas, the adverse effects can be pertained to dose enhancement [124]. Similarly, thymoquinone administration in drinking water at concentration of 0.1, 0.2, 0.3 mg/ml for 3 months to mice revealed no mortality and toxicity and were proved to be safe [125].

In this review, we reported that inadequate literature is available concerning the toxicology and pharmacology of different medicinal plants and their compounds assessed as scolicidal agents against protoscoleces of *E*. *granulosus*. Further research should be conducted to evaluate the toxicology and pharmacology of those plants and compounds possessing promising scolicidal activity.

## Conclusion and recommendations

Recently, research has been flourished and researchers are constantly working on plant extracts and essential oils to find out compounds with high scolicidal efficacies that could be used for the treatment of CE either in combination with or as a replacement for synthetic drugs. Firstly, the benefit of using natural compounds instead of synthetic is that there are fewer chances to develop resistance because there is commonly a mixture of various active compounds having different mechanisms of action. Secondly, due to anthelmintics resistance, the subsequent development of new anthelmintics is very time consuming, and requires a tremendous effort and money. Though, mostly research conducted to control CE via natural products comes to an end in the laboratory because it is very difficult to obtained the same efficacy in the field. Other major obstacles in commercializing an active compound are safety for humans, development of resistance, stability, the probability of synthesis at a reasonable cost as well as environmental safety. It is also concluded from the study that the market for plant-based scolicidal agents is very promising, particularly if the increasing number of side effects of synthetic scolicidal agents are considered. Based on the findings in this review, the following suggestions are recommended.

- Among all plant parts, leaves are widely studied for scolicidal activities, other plant parts should also be investigated for active compounds against CE.
- In future active phytochemicals of plants with high efficiency against *E*. *granulosus* should be investigated separately, that would be helpful to identify and quantify the efficacy of each and every individual compound.
- Among the pure compounds isolated from plants reported in this review, thymoquinone showed a remarkable *in vitro* scolicidal activity with a minimum concentration of 1 mg/ml and 1 min of exposure time. However, its toxicity levels and *in vivo* efficacy has not been documented yet. It is highly recommended to further exploit the efficacy of this compound using *in vivo* models and its toxicity levels in the future.
- The mechanisms of action of plant-based compounds should be investigated in order to thoroughly understand and make improvements in the pharmacological and therapeutic properties of these compounds.
- Most of the plant’s scolicidal activities have been tested by *in vitro* studies, only few studies reported the *in vivo* efficacy of medicinal plants, it is recommended to explore the *in vivo* biological activities of different plants against *E*. *granulosus* to understand more in-depth scolicidal efficacy.
- The knowledge about the toxicity of the plants reviewed in the article is very scarce, hence, further research should be carried out to exploit the toxicity levels of various plants and their active components.
- Most of the medicinal plants and their compounds are evaluated against the common G1 genotype of *E*. *granulosus* focus should be given to other genotypes of *E*. *granulosus* in order to thoroughly understand the efficacy of medicinal plants against other genotypes.
- More focus should be given on parasitocidal rather than parasitotatic (or anti-parasitic) activities of medicinal plants and their constituents.
- More than 68.3% of pharmacological studies are carried out in Iran while the remaining 32.0% in other countries of the world, hence researchers are invited from all over the world to explore the medicinal plants against *E*. *granulosus* for the development of novel and cost effective scolicidal agent to control this zoonotic helminthiasis.

## Supporting information

S1 Table PRISMA checklist. (DOCX)
